# The Influence of Coronavirus Diseases 2019 (COVID-19) Pandemic and the Quarantine Practices on University Students' Beliefs About the Online Learning Experience in Jordan

**DOI:** 10.3389/fpubh.2020.595874

**Published:** 2021-01-13

**Authors:** Ensaf Y. Almomani, Ahmad M. Qablan, Fatin Y. Atrooz, Abbas M. Almomany, Rima M. Hajjo, Huda Y. Almomani

**Affiliations:** ^1^Department of Pharmacy, Alzaytoonah University of Jordan, Amman, Jordan; ^2^Department of Secondary Education, University of Alberta, Edmonton, AB, Canada; ^3^Department of Pharmacological and Pharmaceutical Sciences, College of Pharmacy, University of Houston, Houston, TX, United States; ^4^Department of Pharmacy, University of Lincoln, Lincoln, United Kingdom

**Keywords:** COVID-19, quarantine, online learning, Jordan, knowledge, university students, pandemic, distance learning

## Abstract

Coronavirus Disease 2019 (COVID-19) is a contagious disease that affects the respiratory system. In addition to the severe effects of the disease on health, the pandemic caused a negative impact on basic needs and services, employment, education, and economy worldwide. In Jordan, the whole country locked down, and quarantine was enforced by the military forces, which successfully controlled the spread of the disease. This research aims to study the influence of the COVID-19 pandemic and its associated quarantine on university students' beliefs about online learning practice in Jordan. An online descriptive survey involved questions that covered students' demographic information, student's basic and advanced knowledge about COVID-19, students' online learning experience during the quarantine, and finally students' views on the enforced quarantine practice in Jordan. Results showed that students have a good knowledge (>50%) about the COVID-19 basic information and a moderate knowledge (<50%) regarding COVID-19 advanced information. In general, students were pessimistic about the future of COVID-19 both locally and worldwide. Although some students acknowledged that they learned new skills in the fields of electronics, informatics, and computer software during the pandemic, most of them were unsatisfied about the quality and quantity of the given material, online exams, and the evaluation processes. Unfortunately, most of the students faced internet technical problems or challenges to electronic accessibility. The majority of the participants (>90%) supported the military-enforced quarantine implemented in the country despite the hard time the students had during the quarantine. We conclude that university students were able to protect themselves from COVID-19 through their good knowledge about the infectious disease and their commitment to follow the rules imposed by the Government of Jordan. Nevertheless, the challenges caused by the pandemic and its associated quarantine, combined with the sudden unprecedented online experience, negatively impacted students' thoughts and beliefs about the online learning experience during the quarantine. Further studies need to be performed in this context. We hope our results will help decision-makers better understand the students' attitudes and motivation toward online learning and how this will affect their future plans and decisions.

## Introduction

Coronavirus disease 2019 (COVID-19) (1) is a new infectious disease that emerged in late December 2019, apparently in Wuhan, China. The disease is caused by a novel virus, SARS-CoV-2, which belongs to the *Coronaviridae* family of viruses (2). The local disease then spread worldwide to reach more than 200 countries, causing a pandemic. On March 11th, the World Health Organization (WHO) has announced COVID-19 as a global pandemic (3). COVID-19 is a highly contagious disease that affects the respiratory system. It spreads by aerosols which could stick and contaminate any surface and remain viable for several days (4, 5). COVID-19 symptoms range from mild to severe; it could be similar to mild flu with fever, dry cough, shortness of breath, and fatigue symptoms. In 20% of the infected patients, the disease may develop to become a more serious systemic disease with more serious complications like breathing difficulties, pneumonia, and heart and kidney failure in high-risk patients like old people and those with comorbidities such as hypertension, diabetes, cancer, and heart problems (6, 7). The SARS-CoV-2 is a positive-sense single-stranded RNA virus; it shares 96.2% genomic similarities with the bat coronavirus COV RaTG13 (8) and 79.5% similarities with the SARS-CoV that caused the Severe Acute Respiratory Syndrome (SARS) outbreak in 2002 (9). It started in China and has spread to 17 countries infecting over 8,000 people with a high mortality rate of 9.6% (10). A similar disease is called Middle East Respiratory Syndrome (MERS) caused by the MERS-CoV virus, which also belongs to the same family of coronaviruses. The disease started in Saudi Arabia in 2012, spread to 21 countries, and infected 2,506 people with a 34.0% mortality rate (11). As of December 4th, 2020, according to the WHO, COVID-19 has infected 64,350,473 people with 1,494,668 confirmed deaths worldwide. The highest number of infected people was in the Americas, followed by Europe, Southeast Asia, Eastern Mediterranean, Africa, and the Western Pacific. Unfortunately, despite the hard work in controlling the spread of COVID-19, all attempts to prepare a vaccine or to find an antiviral drug for the virus have not worked yet (12, 13).

Jordan is one of the countries affected with the COVID-19 pandemic. The country took early cautious steps that significantly helped in controlling the spread of the disease. In the middle of March 2020, Jordan declared a national defense law to minimize the spread of COVID-19 in the country (14). A few days later, a military-enforced quarantine was put into effect throughout the whole country, with a curfew that restricted individuals and car mobility. Additionally, all the national institutions including schools, universities, recreation centers, coffee shops, grocery stores, airports, and borders were closed until further notice. Only hospitals and vital centers were allowed to open during the curfew (15). According to the Jordanian Ministry of Health, there were 234,353 infected individuals and 2,960 confirmed deaths in Jordan by December 4th, 2020. Not until June 4th, 2020, when Jordan announced the end of the quarantine, were people allowed to gradually resume their work. Nevertheless, many entities remained closed including wedding halls, condolence houses, youth summer camps, cinemas, public parks, play areas, entertainment centers, schools and kindergartens, and universities and colleges. The quarantine lasted for almost 3 months, during which university students had to stay at home and continue their education through online teaching. A new teaching and learning experience impacted students, instructors, and even families in Jordan.

The COVID-19 pandemic introduced structural changes in teaching and learning strategies. University students continued their education during the pandemic through online learning platforms. However, online learning outcomes varied in different countries. They were affected by online learning infrastructures like internet accessibility and speed and the availability of electronics and computers. Several techniques were used to evaluate the online learning platforms during the COVID-19 pandemic like the online photovoice methodology (a community-based qualitative research approach in which participants take photographs and compose voices) (16) to advocate the facilitators and barriers for online learning during the COVID-19 (17) and to study the economical and psychological impact on students' mental health during the pandemic (18).

This research aimed to explore students' knowledge about COVID-19, the impact of quarantine practices on students' life, and students' beliefs about the online learning that they received during the pandemic using an online structured questionnaire. It is expected that the outcomes of this study will help decision-makers understand students' attitudes and motivation toward online learning and how this will affect their plans and decisions.

## Research Questions

The main purpose of this study was to explore the impact of the COVID-19 pandemic quarantine on students' general knowledge, online learning, and beliefs in Jordan, to help policymakers understand their attitudes and what motivates them about online learning.

The first part of the study was about the students' knowledge and future beliefs about the COVID-19 pandemic. It answered the following questions:

1. What sort of knowledge do university students have about the COVID-19 pandemic?
2. How did the students express their commitment toward reducing the spread of the COVID-19 disease?
3. What are students' beliefs about the future of COVID-19?

The second part of the study highlighted the online learning experience the students received during the COVID-19 pandemic quarantine. It answered the following research questions:

1. What are students' beliefs and thoughts about online learning during the COVID-19 pandemic?
2. What are the links between participants' beliefs and gender, age, field of study, and academic year?
3. What sort of skills related to electronics and computer software enriched students' learning during the COVID-19 pandemic?
4. What are the limitations of online learning from university students' perspectives?
5. What are students' beliefs about the enforced quarantine practices during the COVID-19 pandemic?

## Methodology

### Study Design

An online survey was performed to study the effect of the COVID-19 pandemic and its associated quarantine practices on university students between the period of May 10th and June 6th, 2020. A survey questionnaire was prepared in English and then translated into Arabic (the native language of our respondents), so it will be easier to fill by the students who are not familiar with some English terminology used in the questionnaire. The translation was done first by using the online Google Translate program, and then it was edited by a bilingual university faculty member. The survey questionnaire was prepared in Google forms, which is easy to be filled by the students regardless of their educational levels or majors. The survey link was distributed to the academic staff in several Jordanian universities and was sent to the university students' emails. It was also posted on university-related official Facebook pages to maximize the reach to students of different majors.

### Sample Size Calculation

In this study, the minimum sample size was calculated using the online Raosoft sample size calculator (19), with a confidence interval of 95% and a 5% margin of error. According to the Ministry of Higher Education and Scientific Research in Jordan, the university students' population in the year 2019 was 342,104 students in both public and private Jordanian universities. Based on this, we considered this number (342,104) as the whole population for our study. Accordingly, the calculated minimum sample size is 384 students. We collected 585 responses with a return rate of 11%.

### Survey Validation and Reliability

The first page of the survey included introductory information. The introduction explained the study's purpose and domains. It also involved informed consent statements regarding participation in the study and confidentiality of the data. Students were explicitly informed that they are free to participate in the study. Additionally, the students' participation will be anonymous. Moreover, their answers will be confidential and will not affect their academic marks or their relationships with their instructors. The Cronbach alpha of the instrument was 0.70, which indicated an acceptable consistency level of the survey items. Students were informed that the survey takes 10–15 min to be filled based on the surveyor's comments. The survey was evaluated and approved by the Institutional Review Board (IRB) Committee of Al-Zaytoonah University of Jordan.

### Flow of the Survey Questions

This survey consisted of about 30 questions, where the first group of questions covered socio-demographic information. It included questions about students' gender, age, academic field, academic year, study program, and whether the student is a national or international student in Jordan. The second group of questions explored students' knowledge, commitments, and future beliefs about the COVID-19 pandemic. It discussed students' knowledge about the COVID-19 pandemic and quarantine, students' commitment toward reducing the spread of the COVID-19 disease, and students' beliefs about the future of the COVID-19 pandemic.

The third group of questions discussed the online learning experience of students during the COVID-19 pandemic and quarantine. It asked about the learned skills related to electronics and computer software that enriched students' learning processes during the COVID-19 pandemic, students' beliefs about online learning during the COVID-19 pandemic, and the online learning limitations according to the university students' point of view.

### Statistical Analysis

Data were analyzed using the Statistical Package for Social Sciences (SPSS) software. Descriptive statistical measures and indices were calculated to answer the research questions.

## Results

### Socio-Demographic Information of the Participants

**Figure 1:**
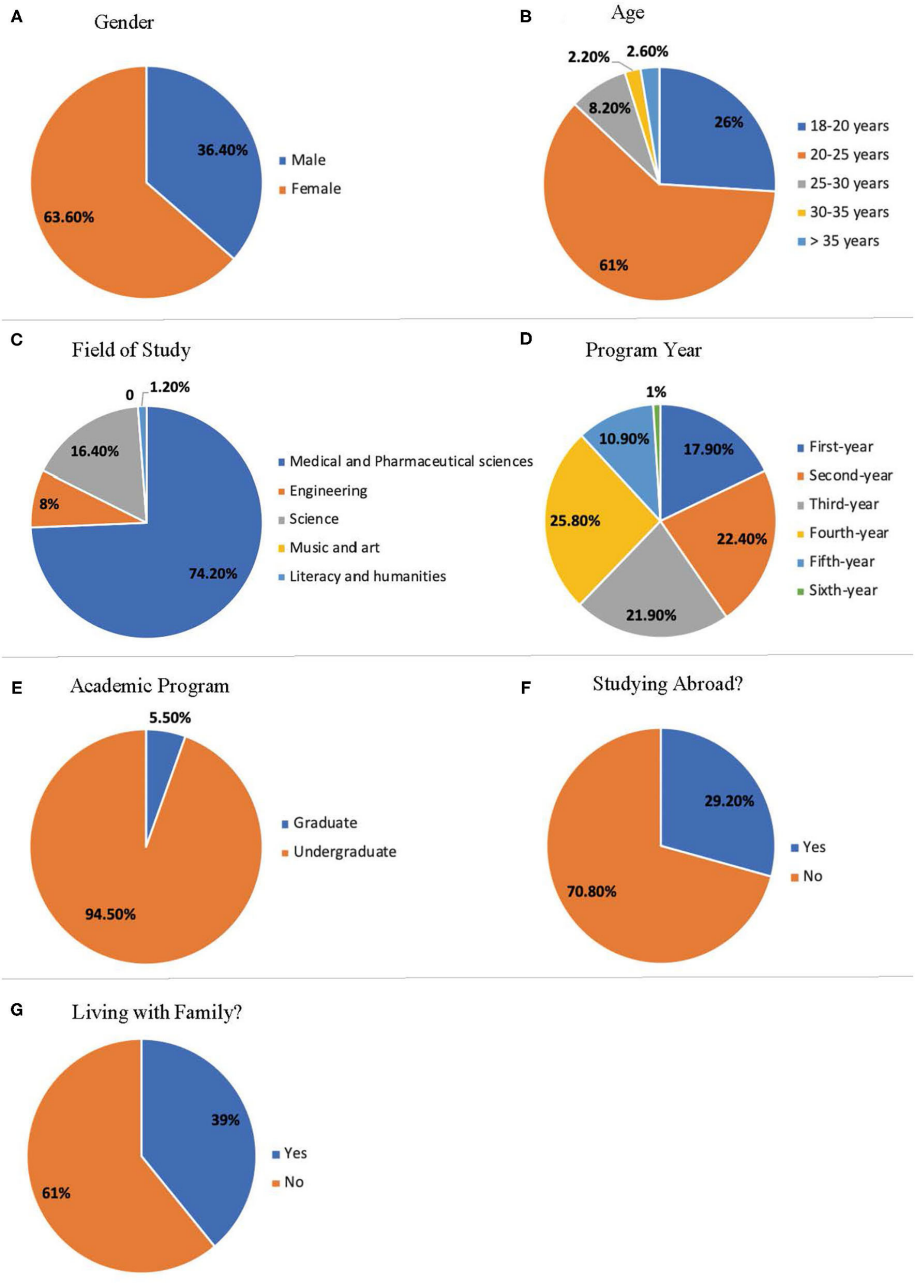
Socio-demographic information of the study participants. **(A)** Participants' gender. **(B)** Age of the participants. **(C)** Field of study of the participants. **(D)** Academic program year. **(E)** Academic program of the participant. **(F)** Percentage of the international students who are studying in Jordan. **(G)** Percentage of the students who are living with their families.

Among the 585 students who participated in this study, 372 (63.6%) were females (Figure 1). The largest category of the surveyors is studying medical and pharmaceutical sciences (434, 74.2%), followed by general sciences (96, 16.4%), engineering (47, 8%), and literacy and humanities (7, 1.2%) (Figure 1). Most of the students are aged between 18 and 25 years old (Figure 1). The surveyors were almost equally distributed among the first, second, third, and fourth academic years (Figure 1). Most of the surveyors, 553 (94.5 %), are enrolled in undergraduate programs (Figure 1). Thirty percent of the surveyors are international students who are studying in Jordan away from their home country (Figure 1). Of the surveyors, 228 (39%) were studying and living away from their families during the quarantine (Figure 1).

### Students' Knowledge, Commitment, and Future Beliefs About the COVID-19 Pandemic

#### What Sort of Knowledge Do University Students Have About the COVID-19 Pandemic?

Participants were asked several questions to assess their knowledge about the COVID-19 pandemic. Almost 95% of the participants kept themselves updated about COVID-19 news during the quarantine. To get more insights about the sort of knowledge the university students hope to learn during the pandemic, we asked them to choose the field(s) they are interested to learn more about regarding the COVID-19 disease (Table 1). About 56% were interested in COVID-19 spread and ways of protection from its infection (Table 1). Most of the surveyors (67.2%) cared about the successful attempts toward finding a vaccine and treatment for the coronavirus. Besides, 54% followed the governmental policies and strategies made to deal with the COVID-19 pandemic in Jordan (Table 1). Furthermore, 42.1% of the surveyors were following global news about the COVID-19 pandemic in different countries, and 47% were concerned about the death and recovery rates of the COVID-19 pandemic locally and worldwide (Table 1).

**Table 1:** University students' knowledge assessment questions and percentages.

**Item**	**Response**	**%**
Do you keep yourself updated about the COVID-19 pandemic?	Yes	94.7
	No	5.3
What field(s) have you aimed to learn more about regarding the pandemic (you can choose more than one)?	1. The spreading methods and protection techniques against the COVID-19 infection	56.1
	2. The successful attempts toward finding a vaccine and treatment for the novel virus	67.2
	3. The governmental policies to deal with the COVID-19 pandemic	54.2
	4. The global news about the COVID-19 pandemic in different countries	42.1
	5. The death and recovery rates of the COVID-19 pandemic locally and worldwide	47
Do you think that the COVID-19 virus infection has a serious effect on health?	Yes	87.4
	No	12.6
Are you aware of the different ways to spread the COVID-19 disease?	Yes	93.5
	No	6.5
Are you committed to following the safety rules to minimize the virus spread like social distancing and wearing of protective masks?	Yes No	94.4 5.6
Do you think that the severity of the COVID-19 infection is race specific?	Yes	12.5
	I don't know	41.2
	No	46.3
Do you think that the severity of COVID-19 infection is gender specific?	Yes	5
	I don't know	59.7
	No	35.4
Do you think that the severity of the COVID-19 infection is affected by the weather and the general climate?	Yes	35.2
	I don't know	30.8
	No	34
Do you think that the severity of the COVID-19 infection is age specific?	Yes	74.5
	I don't know	8.7
	No	16.8

Concerning their knowledge regarding the effect of the COVID-19 disease on health, majority of the students (87.4%) think that the COVID-19 virus infection has a serious effect on health (Table 1). Moreover, most of the surveyors (93.5%) showed a high degree of awareness about the ways of spreading of the COVID-19 disease (Table 1). They also showed a high degree of commitment (94.4%) toward following the safety rules to minimize the virus spread including social distancing and wearing personal protective equipment (PPE) (Table 1).

To get an insight into students' specific knowledge regarding the severity of COVID-19 disease, we asked whether the disease is race specific, gender specific, or age specific: 12.5% of the surveyors thought the disease is race specific, while 5% said that the COVID-19 is gender specific. Interestingly, 74.5% of the surveyors stated that the COVID-19 is age specific (Table 1). Furthermore, 35.2% thought that the spread of COVID-19 disease is affected by the weather and the general climate.

#### How Did the Students Express Their Commitment Toward Reducing the Spread of the COVID-19 Disease?

**Figure 2:**
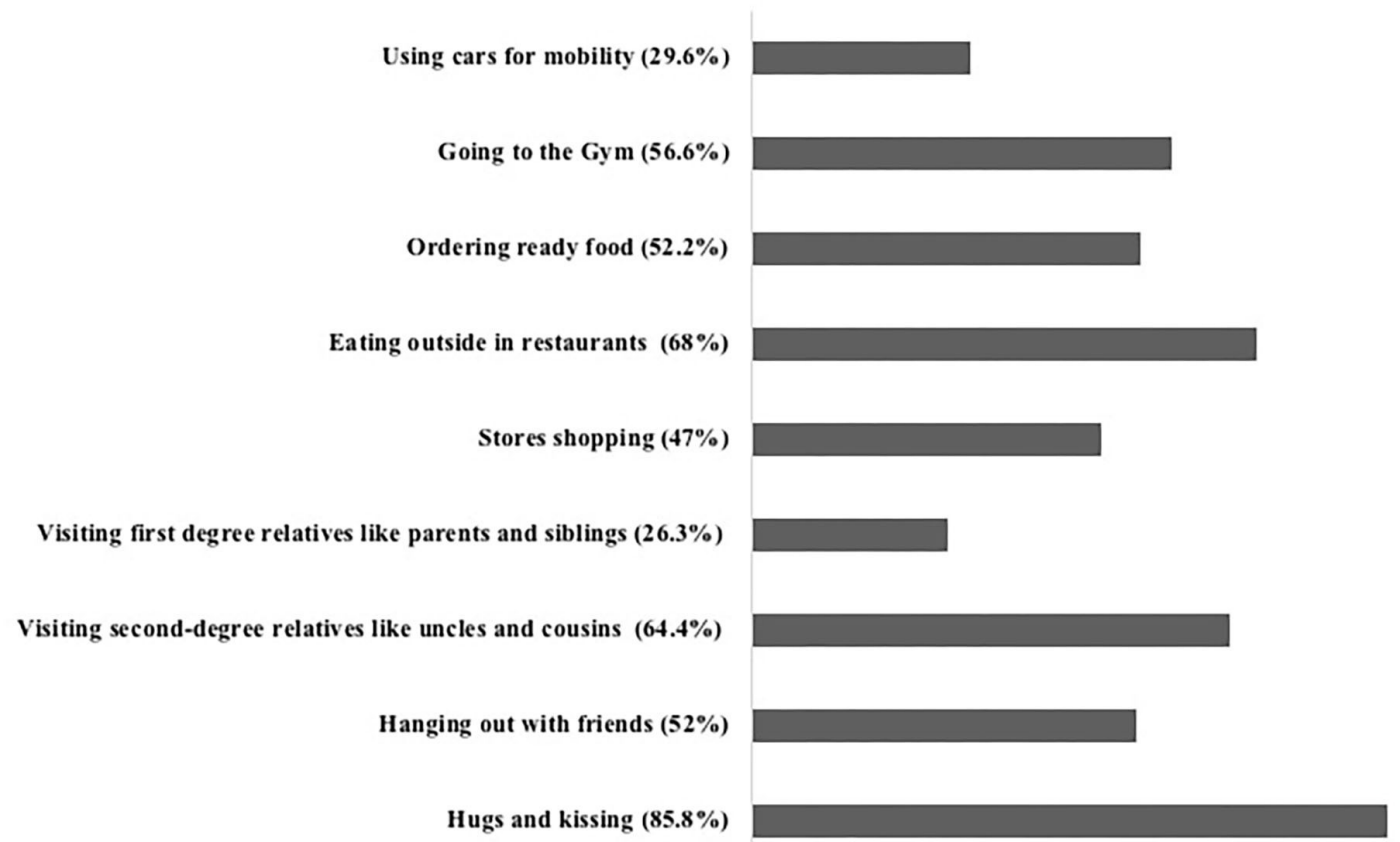
Life routines and habits that students are willing to change to control the spread of the COVID-19 disease.

We were interested to know how students participated in reducing the spread of COVID-19 by asking them about their life routines and habits they are willing to do to control the spread of the COVID-19 disease. Hugging and kissing are used very often in Arab countries as a type of greeting. Surprisingly, 85.8% of the surveyors stopped hugging and kissing for greetings to reduce the disease spread (Figure 2). About 52% stopped hanging out with their friends during the pandemic (Figure 2), and 64% were ready to stop visiting second-degree relatives like uncles and cousins (Figure 2). When we asked students if they are going to stop visiting their first-degree relatives like parents and siblings, only 26.3% of the surveyors would not mind doing so to stop the spread of the disease and to protect their loved ones (Figure 2). Since shopping could contribute to increasing the chance of COVID-19 infection, nearly half (47%) of the surveyors did not mind stopping store shopping (Figure 2), and large shares of the participants (68% and 52.2%) were ready to stop eating in restaurants and to order food from outside the house, respectively (Figure 2). Of the surveyors, 56.6% stopped going to the gym. Interestingly, only one third (29.6%) of the students were willing to stop using cars for mobility during the pandemic (Figure 2).

#### What Are Students' Beliefs About the Future of COVID-19?

We were also keen to know students' thoughts about the future of the COVID-19 disease (Table 2). The students' responses showed that they were worried about the future of the disease. Only 22.4% thought the pandemic will end before the end of 2020. Of the students, 22.7% believed that there could be a second wave of COVID-19 pandemic on the next flu season (Table 2). The surveyors were not very optimistic about finding a vaccine for the COVID-19, and the majority (56.2) was not sure about finding a vaccine before the next flu season (Table 2).

**Table 2:** Students' beliefs about the future of the COVID-19 pandemic.

**Item**	**Response**	**%**
Do you think that the COVID-19 pandemic will end before the end of this year?	Yes	22.4
	Maybe	55.9
	No	21.7
Do you think there will be a second wave of COVID-19 pandemic on the next flu season?	Yes	22.7
	Maybe	60.2
	No	17.1
Do you think there will be an available vaccine for the COVID-19 before the next flu season?	Yes	28.9
	Maybe	56.2
	No	14.9
Are you optimistic about the future of the COVID-19 disease in your country?	Yes	42.9
	I don't know	43.4
	No	13.7
Are you optimistic about the future of the COVID-19 disease worldwide?	Yes	21.9
	I don't know	56.4
	No	21.7

Lastly, we asked for the students' opinions about the future of the COVID-19 disease in Jordan and worldwide. Around 43% of them had positive thoughts about the future of the COVID-19 disease in Jordan, while only 21.9% had positive thoughts about the future of the COVID-19 disease worldwide.

### Online Learning Experience During the COVID-19 Pandemic Quarantine

#### What Are Students' Beliefs and Thoughts About Online Learning During the COVID-19 Pandemic?

During the curfew which lasted for 3 months in Jordan (15), all university students continued their education online. This section discussed the online learning experience during the COVID-19 pandemic and quarantine.

First, the students were asked to assess the quality of the online learning experiences they received during the COVID-19 pandemic compared to the face-to-face learning on campus. Unexpectedly, the majority of the participants (80.3%) think that the quality of learning was decreased, 9.6% mentioned that it increased, and 10.1% answered it was not affected (Table 3). According to the students, the online education quantity was also affected: 36.4% said the online education quantity was increased, 19.8% answered it was not affected, and 43.8% answered it decreased (Table 3). Next, we asked the students what they think of the online learning strategy followed by their institute during the COVID-19 pandemic: 6.5% answered by excellent, 20.5% said very good, 41.7% said satisfactory, and 31.3% said it was below expectations (Table 3).

**Table 3:** Students' beliefs and thoughts about online learning during the COVID-19 pandemic.

**Item**	**Response**	**%**
What do you think about the online education quality during the COVID-19 pandemic compared to the school education?	Increased	9.6
	Not affected	10.1
	Decreased	80.3
What do you think about the online education quantity during the COVID-19 pandemic compared to the school education?	Increased	36.4
	Not affected	19.8
	Decreased	43.8
What do you think about the online learning strategy followed by your institute during the COVID-19 pandemic?	Excellent	6.5
	Very good	20.5
	Satisfactory	41.7
	Below expectations	31.3
What is your degree of commitment toward submitting the online homework and quizzes?	100%	71.5
	75%	19.3
	50%	5.1
	25%	4.1
What do you think about the online exams compared to the in-campus exams?	More preferred	16.8
	No difference	15.2
	Less preferred	68.0
What do you think about the evaluation process used by your campus during the coronavirus pandemic quarantine?	Fair	38.5
	Not fair	61.5
In the coming future, will you register in online classes if available at your institute?	Yes	23.6
	Maybe	28.2
	No	48.2

To get an idea about students' dedication and involvement in the online learning, we asked how committed they were toward submitting their online homework and quizzes, and we asked them to choose either submitting 100, 75, 50, or 25% of the given homework. The majority of the participants (71.5%) submitted 100% of their online duties, 19.3% submitted 75, 5.1% submitted 50, and 4.1% submitted 25% (Table 3).

The online learning experience is very new to most of the students in Jordan; therefore, we asked them if they prefer the online exams over the face-to-face on-campus exam: the majority of the students (68%) said they are less preferred, 15.2% answered they had no difference, and only 16.8% answered they are more preferred (Table 3).

Online teaching and learning were combined with online evaluation through submitting homework and quizzes. The instructor had to evaluate the students' effort based on what they submitted of the given homework and on their effective communication with their instructors.

We asked the students to state their opinion about the evaluation process used by their institution during the COVID-19 pandemic quarantine. Although the online evaluation during the quarantine gave the students the chance to have higher marks compared with the on-campus evaluation, the majority of the students (61.5%) said it was not fair, while 38.5% answered it was (Table 3).

The online teaching process is quite new in Jordan, so we opted to ask the students if they will register to online classes if available at their institutions in the coming future; 23.6% answered yes, 28.2% answered maybe, and 48.2% answered no (Table 3).

#### What Are the Links Between Participants' Beliefs and Gender, Age, Field of Study, and Academic Year?

We further analyzed the students' beliefs about online learning during the pandemic based on gender, age, fields of study, and academic year using the SPSS statistics program. We found that 66.0% of the female students were satisfied with the online learning strategy followed by their institution during the COVID-19 pandemic, which is significantly higher than the rate (34%) of males who reported their satisfaction. We also found that 70.6% of the female students were more committed to submitting all their homework and quizzes as compared to males (29.4%). Furthermore, female students were also more optimistic (70.2%) regarding the evaluation process followed by their institute during the coronavirus pandemic quarantine than are males (29.8%). When we analyzed the data based on the academic year, it was quite obvious that students from the second to the fourth years are more able to deal with online learning than are first-year students.

#### What Sort of Skills Related to Electronics and Computer Software Enriched Students' Learning During the COVID-19 Pandemic?

**Figure 3:**
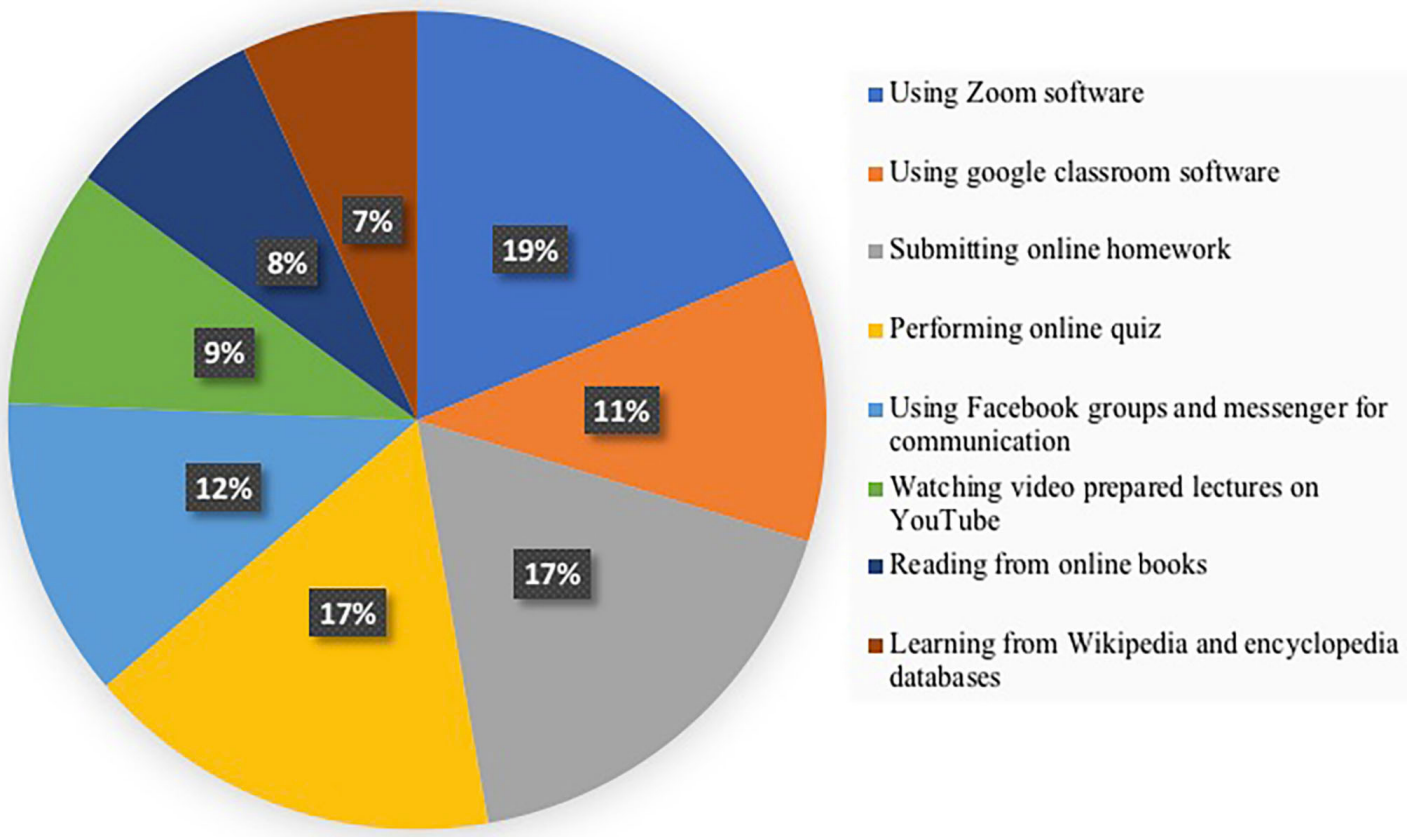
Electronics and software skills that enriched students' learning during the COVID-19 pandemic.

Despite the fact that many students were not satisfied with the online teaching and learning process, we opted to ask the students about the newly learned skills in the field of electronics and computer software that enriched and expanded their knowledge in their fields of study. We found that 71.3% of the students learned how to use the Zoom software (the video conferencing tool) to attend online classes (Figure 3), 42.9% practiced using the Google Classroom software (Figure 3), 66.8% learned to submit online homework (Figure 3), 63.4% learned how to perform online quizzes and exams (Figure 3), 45.6% started using Facebook groups and Messenger for communication with their instructors and colleagues (Figure 3), 36.2% watched video-prepared lectures on YouTube that are related to their courses (Figure 3), and 30.4% read from online books to get the most outcome of their classes (Figure 3). Lastly, 26.7% stated that they obtained information and knowledge from Wikipedia and encyclopedia databases (Figure 3).

#### What Are the Limitations of Online Learning From University Students' Perspectives?

**Figure 4:**
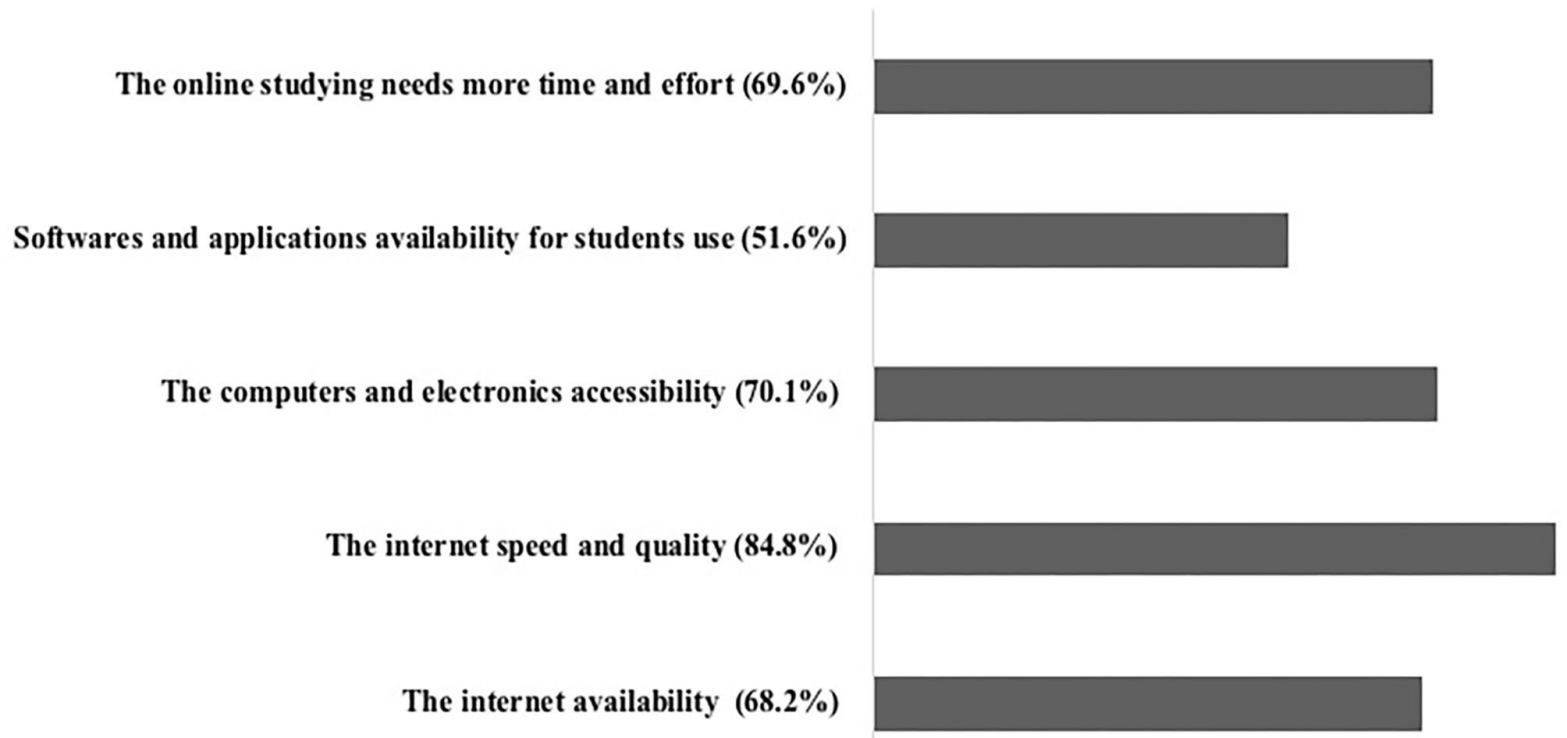
The online learning limitations during the quarantine.

Notably, the students were asked to state the limitations that affect online learning outcomes. According to them, the main online limitations as represented in Figure 4 are the following: 68.2% said the availability of the internet, 84.8% thought the internet speed and quality, and 70.1% believed it is the computers and electronics accessibility. Additionally, 51.6% answered the availability of different applications and software the student needs to use. Moreover, 69.6% thought they need to invest more time and effort in online studying compared to the on-campus learning.

One of the most important learning limitations during the pandemic was the students' financial situation. We asked the students if they were financially stable during the pandemic. Around 44% reported that they were financially stable, while 55.4% reported that they were not.

#### What Are Students' Beliefs About the Enforced Quarantine Practices During the COVID-19 Pandemic?

In this part, we wanted to study the effect of the quarantine resulting from the COVID-19 pandemic on students' life routines and state of feelings. First, we asked the students if the enforced quarantine has a positive impact on their lives (Table 4). A large share (80%) agreed that the enforced quarantine minimized the number of affected individuals. A great majority of the surveyors (89%) thought that the quarantine controlled the disease spread in the country, 47.9% stated that the enforced quarantine helped in curing the affected patients faster, and 65.1% thought it helped in isolating the affected regions efficiently. Although the Jordanian people are very social and welcoming with tied family bonds, nearly half of the students (49.6%) thought that the enforced quarantine mitigates the intimate way of greetings that involve hugging and kissing.

**Table 4:** Students' beliefs about the enforced quarantine practice during the COVID-19 pandemic.

**Item**	**Responses**	**%**
In your opinion, the COVID-19 quarantine benefits are:	1. Minimize the number of affected individuals	80
	2. Control the spreading of the disease	88.9
	3. Help in curing the affected patients faster	47.9
	4. Help in isolating the affected regions	65.1
	5. Replacing and minimizing some social habits that are extremely used in our community like hugging and kissing	49.6
	6. Learning new skills	38.3
	7. Having more family time than usual	44.3
The early home quarantine strategies followed in Jordan contributed in preventing further spreading of the disease, what do you think about this?	Strongly Agree	51.8
	Agree	38.5
	Neutral	7.9
	Disagree	1.9
Do you think that the quarantine for either the healthy people at home or the affected people at hospitals is the most important and effective step in preventing the spreading of the disease?	Yes No	92.3 7.7
Do you support the military-enforced quarantine?	Yes	94.7
	No	5.3

Interestingly, some of the participants were optimistic about the benefits of the quarantine: 38.3% of the participants thought that quarantine gave them extra time to learn new skills, 44.3% stated that they had more family time than they used to have before the quarantine started, and 33.8% of the surveyors changed their diet into more natural food to boost their immune system (Table 4).

Even with one case of COVID-19 infection on March 14th, 2020, the Jordanian government implemented a strict military-enforced quarantine early during the pandemic (14). All recreation centers, grocery stores, public and private transportations, and even the government offices were closed for a few months before they started a gradual reopening (14). We asked the participants what they think about the early home quarantine strategies followed in Jordan to control the COVID-19 disease spread. Around half of them (51.8%) strongly agreed with those strategies: 38.5% agreed with them, 7.9% were neutral, and only 1.9% disagreed with the home quarantine strategy (Table 4). Then we asked the students what they think about enforcing healthy people to stay at home, and the affected people at hospitals as a precautious step to minimize the spread of the disease. Most of the surveyors (92.3%) agreed. Finally, we asked students if they supported the military-enforced quarantine performed in Jordan. The vast majority of the students (94.7%) answered by yes (Table 4).

## Discussion

This is a descriptive online survey discussing the influence of the COVID-19 pandemic and quarantine on students' general knowledge, academic life, and beliefs. To our knowledge, this is the first study that explored students' beliefs about the online learning experience during the COVID-19 pandemic and quarantine time in Jordan. Previous studies either focused on knowledge and information in medical and non-medical students (20) or discussed the psychological impacts of the COVID-19 pandemic on university students (21). Most of the participants in our study were undergraduate females, aged between 20 and 25 years, studying medical and pharmaceutical sciences (Figure 1).

The first part of this study discussed students' knowledge, commitment, and future beliefs about the COVID-19 pandemic. First, we asked about the sort of knowledge university students have about the COVID-19 pandemic. To answer this question, we asked the students questions to describe their general knowledge about the COVID-19 news updates, ways of spreading, ways of protection, and effect on health. We found that most of the students were well-informed and educated about the COVID-19 disease as 95% of the participants kept themselves updated about the COVID-19 pandemic. Our results are consistent with a previous study by Olaimat et al. that described the knowledge and information about the COVID-19 among university students in Jordan; the study found that the average knowledge scale of Jordanian students was 80.1%, which is considered good according to the knowledge scale (20). Another early study during the pandemic was conducted by Alzoubi et al. about the COVID-19 knowledge, attitude, and practice among university students, in which 90% revealed a positive response regarding overall knowledge about the symptoms of the COVID-19 and 99.7% had a good attitude and practice toward protecting themselves from infection (21).

In our study, we attempted to get an insight into students' specific knowledge regarding the severity of COVID-19 disease. Except for age, students' answers showed low to moderate (<50%) knowledge about the link between COVID-19 disease severity and patients' gender, race, and the effect of weather and general climate. Statistical analysis of the fatality rate and risk factors showed that the risk of death associated with COVID-19 infection is higher in old people aged >60 years (22). Additionally, men are at higher risk of death associated with the COVID-19 disease as compared to women (23). Interestingly, a recent update by the Centers for Disease Control and Prevention (CDC) showed a correlation between race and ethnic minority groups with the increased risk of being infected by the COVID-19 disease. They showed that the non-Hispanic American Indian or Alaska Native were at higher risk of infection, followed by the mon-Hispanic Black, Hispanic and Latino, non-Hispanic Asian, and lastly the non-Hispanic White (1).

The COVID-19 severity had also been linked to the weather; as yet a clear link between high and low-temperature effect and the disease infection rate or severity is absent, though several studies showed a link between the incidence of COVID-19 disease and temperature. A recent study from Indonesia analyzed the effect of different factors like temperature, humidity, and the amount of rainfall on COVID-19 incidence rate, and results showed that the average temperature was significantly correlated with the high COVID-19 incidence rate (24). Another study from 122 cities in China indicated that mean temperature has a positive linear relationship with COVID-19 cases with a threshold of 3°C (25).

To get an insight into students' practices that showed their commitment toward reducing the spread of the COVID-19 disease, we asked them about their life routines and habits they are willing to reduce to control the spread of the COVID-19 disease (Figure 2). Most of the students (≥50%) were willing to reduce most of their habits like hugs and kisses for greetings, visiting relatives, going out, and eating at restaurants. Nevertheless, most of the surveyors (>70%) were still willing to visit their first-degree relatives like parents despite the chance of being infected with the virus. This is not surprising when we take into consideration that the Jordanian community has strong family ties that reflect on their economic outcomes (26).

Pandemics of this kind are always accompanied by the fear of being infected with some mystery about their future. There are no doubts that the daily global news of accelerated COVID-19 infection and mortality rates in addition to the enforced quarantine casted a shadow on people's thoughts and beliefs in regard to the future of the COVID-19 disease (27). Students' beliefs and thoughts about the future of the COVID-19 pandemic were presented in Table 2. Most of the students were pessimistic, with a gloomy and cloudy view about the future of the COVID-19 pandemic at both local and global levels. The stressful environment and the academic pressure might negatively impact their beliefs about the pandemic in the future. Moreover, they were not confident about finding a vaccine for the disease in the coming future. Regardless of all the efforts and attempts to find a vaccine for this virus, there is still no available vaccine till the time of the writing this paper (28).

The second part of this study focused on the online learning experience during the COVID-19 pandemic quarantine, which is discussed for the first time in Jordan. When the curfew started in Jordan, an online educational platform was created by the Ministry of Education in Jordan to allow the teachers to deliver online classes for school students through specific TV channels (29). Also, all higher institutes in Jordan continued their education through online classes. We were interested in students' thoughts and beliefs regarding online learning during the COVID-19 pandemic and quarantine. It was found that online learning has more advantages over paper-based examination in terms of reliability of the grading method, time efficiency, and cost (30). However, our results showed that students were unhappy with the quality and quantity of their online learning experience (Table 3). Answers of satisfactory to below expectations dominated the students' responses when they were asked about the online learning strategy followed by their institute during the COVID-19 pandemic (Table 3). They also showed a negative response regarding the online exams and the evaluation process during the coronavirus pandemic quarantine (Table 3). In agreement with our results, a recent cross-sectional study from the Philippines by Baloran revealed that most of the students expressed negative impressions and disagreed with the conducted Online-Blended Learning Approach during the COVID-19 pandemic at their institutions (31).

Nevertheless, further analysis of the data based on gender indicated that female students were more optimistic about, satisfied with, and committed to the online learning experience during the COVID-19 pandemic than were males. It is worth mentioning that some studies attribute female students' better satisfaction of online learning experience to the value and interest that female students assign for planning and participation (32). Female students also appear to be better prepared and organized as well as more participative and committed to the learning process than are males. Another possible reason for female students' high satisfaction with online learning experiences could be their abilities to adapt to new teaching environments caused by the COVID-19 pandemic (33).

Beyond question, some crisis turns to be an opportunity to learn new skills. The coronavirus pandemic challenged students, professors, and employers to adapt to the online teaching and learning process. Many organizations including international labor organizations and UNESCO offered free online training courses to teach interested people how to use different online teaching programs in the field of online courses and blended learning (32, 33).

Online learning during the pandemic pushed university students to learn new skills and software in the field of electronics to communicate with professors and to submit online assignments and homework. The newly learned skills include using Zoom, Moodle, and Google Classroom; reading from online books; and using Wikipedia and encyclopedia databases (Figure 3).

Despite the hard efforts of the higher education institutions in Jordan to continue students' education through the quarantine, online learning during the COVID-19 pandemic has many limitations that challenged its outcomes (Figure 4). In addition to the time and effort needed to be invested into online learning, internet availability, speed and quality of internet connection, and electronics accessibility altogether caused a diminution in the amount and quality of the delivered educational materials.

Another factor that affects the learning outcome during the quarantine was the students' financial situation; it affects students' engagement in learning, which in turn affects students learning outcomes and future study decisions (34). Most of the participants (55.4%) reported that they did not have stable financial resources during the quarantine, most likely because of the total country lockdown which lasted for a few months and affected financial service entities and students' income resources (15).

Lastly, we discussed student's beliefs concerning the enforced quarantine practice during the COVID-19 pandemic. Results showed that students had positive thoughts about the importance of the lockdown step in controlling the disease spread. Despite the restrictions of individuals' movement and gathering, the vast majority of the participants supported the military-enforced quarantine and its regulation. The quick spread of COVID-19 worldwide; its contagious nature, ways of spreading, resulting health complications, and preventive strategies; and availability of its vaccine altogether created a stigma about the disease and fear of infection and death, so the high hopes of having a world clean of the COVID-19 disease in the coming future is getting low (1, 35, 36); therefore, the majority of students who participated in our study would rather be in lockdown than be at high risk of infection.

## Conclusion

The majority of the students who participated in this survey showed good knowledge about COVID-19 general information like ways of spreading and preventing infection, while they showed a humble knowledge with regard to the specific information about COVID-19 which requires research and investigation, like the link between COVID-19 severity and patients' gender, race, and weather. Most of the students believed that the best way to control the disease spread is to minimize social activities like gathering, hugs for greetings, and going out. Unpleasant COVID-19 news casted a shadow on students' thoughts and beliefs. Students had pessimistic beliefs about the future of COVID-19 locally and worldwide. Moreover, they were also not confident about finding a vaccine in the coming future. The challenges of the new online learning experience during the pandemic have negatively impacted students' beliefs and thoughts. Students were unhappy about the quality and quantity of the given materials, online exams, and the evaluation process. In addition, technical and connectivity problems including internet speed and availability as well as electronics accessibility were identified as barriers to a successful online learning experience. However, further analysis revealed that female students were more optimistic about, satisfied with, and committed to the online learning experience during the COVID-19 pandemic than were males. In contrast to students' negative thoughts about online learning during the pandemic, most of them agreed that they learned new skills in the fields of electronics and computer software during the pandemic. Jordan followed a planned enforced quarantine to control the COVID-19 disease spread and to minimize the number of infected individuals. Despite the hard times that students had during the quarantine, the majority agreed about the importance of the lockdown and military-enforced quarantine in controlling the infection. Hopefully, the results of this study will help decision-makers understand the challenges that affect students' attitude and motivation toward online learning, in a step to improve the online learning platform. This would also reflect on activating strategies to facilitate the online learning process and to improve the online learning outcomes.

### Limitations

- The number of male participants was lower than that of females.
- The number of participating students of different majors was unequal.
- The participants' number was affected by the students' free time during the online learning and the internet and electronics availability.
- Students' thoughts about online learning were influenced by the pandemic and the fear of infection.

### Implications

- Future studies should discuss the effective teaching methods used for different disciplines during the pandemic.
- Further studies that focus on students' online learning experiences and challenges are needed.
- The efficiency of online learning strategies that were used in different countries during the pandemic needs to be discussed.
- Students' mental health during the pandemic and its impact on students' academic performance need to be addressed.

## Data Availability Statement

The original contributions generated for the study are included in the article/supplementary materials, further inquiries can be directed to the corresponding author/s.

## Ethics Statement

The studies involving human participants were reviewed and approved by the Institutional Review Board (IRB) committee of the Alzaytoonah University of Jordan. This study was approved by the IRB with no need for written consent for students' participation. The ethics committee waived the requirement of written informed consent for participation. Written informed consent was not obtained from the individual(s) for the publication of any potentially identifiable images or data included in this article.

## Author Contributions

EA: survey idea, format and design, and writing of the manuscript. AQ: study design and data analysis. FA, AA, HA, and RH: study design and writing of the manuscript. All authors contributed to the article and approved the submitted version.

## Conflict of Interest

The authors declare that the research was conducted in the absence of any commercial or financial relationships that could be construed as a potential conflict of interest.
